# Parkinsonism and Bipolar Disorder

**DOI:** 10.1111/bdi.12888

**Published:** 2020-01-30

**Authors:** Annemiek Dols, Afina W. Lemstra

**Affiliations:** ^1^ Psychiatry Amsterdam Public Health Research Institute Amsterdam Neuroscience Amsterdam UMC Vrije Universiteit Amsterdam The Netherlands; ^2^ GGZ inGeest Specialized Mental Health Care Amsterdam The Netherlands; ^3^ Neurology Alzheimercenter Amsterdam Neuroscience Amsterdam UMC Vrije Universiteit Amsterdam The Netherlands

Parkinsonism is a frequently encountered clinical feature in patients with bipolar disorder (BD). It is usually attributed to side effects of medication, but can also be a result of concomitant cerebrovascular disease and even an emerging idiopathic Parkinson's disease (PD). In a recent meta‐analysis by Faustino et al published in JAMA Neurology, the association of BD with a later diagnosis of PD was assessed.^1^ Four cohort studies and three cross‐sectional studies reporting data on the likelihood of developing PD in BD vs non‐BD populations were included, with a total of 4.374.211 participants overall. Two of the seven studies were considered to have an elevated risk of bias: one due to inclusion of a specific subgroup of veterans limiting comparability and one due to unreliable data collection (self‐reported diagnoses of PD) and a different source of the control group resulting in limited selection and comparability. In the meta‐analysis excluding these two, the likelihood of a subsequent diagnosis of idiopathic PD in patients previously diagnosed with BD was increased (odds ratio, 3.21; 95% CI, 1.89‐5.45; I^2^ = 94%). Previous studies have suggested that PD is probably more common in BD than in the general population, but this review provides for the first time evidence supporting this notion based on a systematically analyzed, large pooled data set. The strength of this study is that a large number of subjects were included. However, several limitations are to be noted. It is not clear how PD‐diagnoses were established, most data were derived from medical records, both cross‐sectional and longitudinal (retrospective and prospective) data were included in the meta‐analysis leading to variation in data quality. Overestimation of the presence of PD is possible, as the diagnostic coding may not have differentiated PD from other causes of parkinsonism, and cases of atypical parkinsonism may have been included. Furthermore, the age of onset of BD in relation to the development of PD over time is not clear, therefore PD cases debuting with mood symptoms may have been included as patients with BD that developed PD subsequently. In a subgroup analysis longer follow‐up was associated with smaller increase in the risk of PD diagnosis. Bearing in mind the difference in the expected age at onset of BD (around 25 years) and PD (around 55 years) this is remarkable and requires further exploration to see if mostly young subjects have been followed longer or that specifically older late‐onset BD cases have an increased risk for PD.

All together the review and meta‐analysis suggest that patients with BD have a significantly increased risk of developing PD compared with the general population.^1^

This study has both clinical and research implications for BD and PD.

The identification of the underlying cause of parkinsonism in patients with BD is of significant clinical relevance. It is a challenge to determine the underlying cause of parkinsonism (defined by resting tremor, bradykinesia, rigidity and/or postural instability) in BD clinically. Clinical signs such as asymmetric motor features, the presence of resting tremor and hyposmia are less suggestive for drug‐induced parkinsonism, but lack specificity.^2^ Additional imaging of the brain with Computer Tomography or Magnestic Resonance Imaging could rule out cerebrovascular pathology. The most reliable way to differentiate PD from other causes of parkinsonism is dopamine transporter (DAT) imaging by ^123^I‐N‐ω‐fluoropropyl‐2β‐carbomethoxy‐3β‐(4‐iodophenyl) nortropane (^123^I‐FP‐CIT) single photon emission computed tomography (SPECT) as a proxy of nigrostriatal degeneration. An ^123^I‐FP‐CIT scan is generally normal in drug‐induced or vascular parkinsonism.

In patients with BD, longitudinal motor assessments could be recommended to screen for prodromal motor and nonmotor signs of PD. Once patients with BD present with tremor or other symptoms of parkinsonism this cannot simply be attributed as drug‐induced and investigation of PD is recommended. Preferably this should be done by a neurologist and include dopaminergic imaging when in doubt. If these symptoms are a result of side effects, these are often dose‐dependent and lowering lithium levels may reduce symptoms of parkinsonism without losing efficacy, especially in older adults.^2^, ^3^

Probably not all BD patients in which parkinsonism becomes manifest should be assessed for PD promptly. The meta‐analysis suggests that only a small proportion of the BD patients are at risk for developing subsequent PD. Next step would be to investigate which patients are at risk and how they can be identified. It seems logical that age is a factor that should be taken into account since PD usually manifests at older age. Also risk estimates per gender should be investigated since PD is more predominant in males.

Of course, this raises the question of the mechanism behind the link between BD and PD. BD can be seen as a brain disorder that debuts earlier in life and converts to neurodegenerative disorder with ageing. Risk factors for this conversion could be genetic or environmental (head trauma, toxic exposure or unfavorable course of BD with allostatic loading by multiple episodes, substance abuse and psychiatric comorbidities). The authors quote the dopamine dysregulation hypothesis that links BD to PD: manic states may lead to a downregulation of dopamine receptor sensitivity resulting in a depressive episode that is in turn compensated by upregulation resulting in a (hypo)manic episode. Numerous cycles may lead to an overall reduction of dopaminergic activity, the prototypical PD state. Further clinical evidence of dopamine dysregulation is found in the fact that patients with BD in a depressed mood experience more parkinsonism, and that patients with PD experience mood changes related to the on‐time/off‐time phenomena, with mania‐like symptoms in the on‐time phase and vice versa. Moreover, levodopa (dopamine agonist) can be used to increase dopamine levels as a therapeutic agent in PD but is also known to induce (hypo) mania, antipsychotic medication that block dopamine receptors can in turn improve manic symptoms but are also known to increase rigidity and hypokinesia in patients with PD.

A shared pathophysiological mechanism (causing dopamine dysregulation) for BD and PD could explain the overlap in a specific subset of patients. Whether lithium can prime for PD is unknown, but parkinsonism is a known side‐effect. However, dementia rates are lower in individuals exposed to standard or trace‐dose lithium, suggesting that neurodegeneration is not induced by lithium itself. Protective factors could also be identified, such as the use of lithium, known to inhibit excitatory neurotransmitters such as dopamine and to have neuroprotective effects.

Another hypothesis could be that BD is a not only a possible risk factor for developing PD later in life but could also be a prodromal feature of PD. In that light it is worth to perform risk‐analyses of BD in the Lewy body disease spectrum, including also Dementia with Lewy Bodies (DLB). Cognitive impairment is considered a common feature in BD, with a progressive nature in a subgroup leading to increased rates of dementia.^4^ The neurobiological underpinnings of this cognitive impairment remain unknown, but risk factors such as numerous mood episodes, psychiatric admissions, psychotic features, earlier age at onset of BD and cardiovascular burden have been suggested.^5^ DLB is defined by progressive cognitive decline accompanied by parkinsonism, visual hallucinations, cognitive fluctuations, and RBD. There is a large overlap with PD both clinically and pathologically. In contrast to Alzheimer's dementia (AD), memory dysfunction is not prominent and brain‐atrophy on structural imaging is relatively absent in DLB, often resulting is a missed diagnosis of DLB in patients with BD presenting increased mood instability and cognitive decline later in life. It is highly likely that similar risk scores as found in relation to PD could be found in DLB. It is known that pathological processes of neurodegenerative diseases like PD, DLB and AD start decades before clinical symptoms manifest. However, it is increasingly recognized that nonmotor, noncognitive phenomena already present in early stages, but usually at that point are not attributed to neurodegenerative disease. A well‐studied syndrome is REM‐Sleep behavior disorder (RBD). This syndrome usually manifests before midlife. Over 80% of the patients with RBD develop Lewy body disease (PD or DLB) and the syndrome is considered a prodromal sign of these diseases. Further research should be undertaken to investigate the possibility if a subgroup of BD could be at a prodromal stage of Lewy Body Diseases. This would have implications for future treatment strategies when potential disease modifying drugs for Lewy body diseases become available. Further pathophysiological and genetic studies should shed light on biological mechanisms that link BD and Lewy body diseases. Both disease entities are known to have genetic susceptibility and possibly genetic variants could explain the co‐existence of both diseases.

This review on the increased risk for PD in patients with BD urges for a new clinical approach. Tremor or other symptoms of parkinsonism in patients with BD cannot simply be attributed as drug‐induced and investigation of PD is recommended including dopaminergic imaging, especially in older and late‐onset cases.

As for research, BD can be seen as a neuropsychiatric disorder that debuts earlier in life with mood symptoms and associated motor and cognitive symptoms later in life may be the first symptoms of a neurodegenerative course. Risk factors for this conversion could be genetic or environmental.

A close collaboration between neurologists and psychiatrists may open new avenues for management of both BD and PD and research in the neurobiological underpinnings of BD.

## CONFLICT OF INTEREST

There are no competing interests to declare.
